# Transcriptomic analysis of left-right differences in human embryonic forebrain and midbrain

**DOI:** 10.1038/sdata.2018.164

**Published:** 2018-09-04

**Authors:** Carolien G. F. de Kovel, Steven N. Lisgo, Clyde Francks

**Affiliations:** 1 Language and Genetics Department, Max Planck Institute for Psycholinguistics, 6525 XD Nijmegen, The Netherlands; 2 Institute of Genetic Medicine, Newcastle University, NE1 7RU Newcastle upon Tyne, UK; 3 Donders Institute for Brain, Cognition and Behaviour, 6525HR Nijmegen, The Netherlands

**Keywords:** Development, Genetics of the nervous system

## Abstract

Left-right asymmetry is subtle but pervasive in the human central nervous system. This asymmetry is initiated early during development, but its mechanisms are poorly known. Forebrains and midbrains were dissected from six human embryos at Carnegie stages 15 or 16, one of which was female. The structures were divided into left and right sides, and RNA was isolated. RNA was sequenced with 100 base-pair paired ends using Illumina Hiseq 4000. After quality control, five paired brain sides were available for midbrain and forebrain. A paired analysis between left- and right sides of a given brain structure across the embryos identified left-right differences. The dataset, consisting of Fastq files and a read count table, can be further used to study early development of the human brain.

## Background & Summary

In addition to the anterior-posterior axis and the dorsal-ventral axis, the body has a left-right axis. Left-right asymmetry of the internal organs such as the heart and liver is obvious, but the central nervous system is asymmetric as well. The asymmetry of the central nervous system is at least partly uncoupled from the asymmetry of the internal organs, as evidenced from observations of people with reversed organ placement^1,2^.

The earliest observed evidence for structural brain asymmetry in development is a small difference in average volume of the left and right sides of the choroid plexus in human foetuses at 11 post conception weeks (PCW)^3^. From 8 PCW, behavioural asymmetry has been observed: embryos more often moved their right arms than their left arms^4,5^. In a previous study, we showed that human spinal cord and hindbrain have left-right differences in gene expression at stages between 4 and 8 PCW^6^. These observations indicate a well-regulated genetic programme to manage early asymmetrical brain development. However, whether the human midbrain and forebrain also show transcriptional asymmetry at such early stages was unknown prior to generation of the current dataset (previous studies had analyzed foetuses at later stages). In fish, molecular studies have revealed mechanisms and genes that are involved in brain laterality^7^, but it is unknown how much of this is conserved in humans.

Our previous studies^6^ suggested a difference in maturation rate between left and right sides of spinal cord and hindbrain as one mechanism to arrive at left-right differences. Also, we observed that the left-right pattern in the hindbrain was a mirror-image of that in the spinal cord. To further elucidate the left-right differences in the developing human brain, we now dissected midbrains and forebrains of six human embryos, at Carnegie stages 15 or 16, into left and right. The embryos were from social pregnancy terminations. RNA was isolated from each brain side separately. Barcoded cDNA fragments were sequenced on an Illumina HiSeq 4000 sequencer, using paired-end sequencing with a read length of 100 bases. After filtering for quality, the median size was 5.65 Gb per library. Data are made available as Fastq-files and a processed data table containing gene counts. Our analysis of this dataset will be published elsewhere.

## Methods

### Collection, library preparation and sequencing

**Figure 1:**
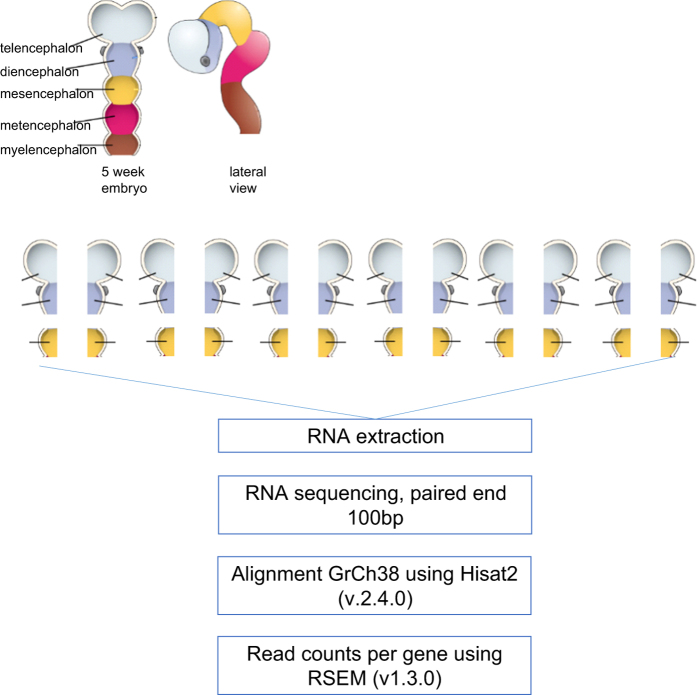
Experimental design. Six human embryos at Carnegie stages 15 or 16. Forebrain and midbrain were dissected and divided into left and right. RNA was isolated from the complete tissue sample. Paired end sequencing (100 bp) was done at BGI, China. Alignment and read counts were performed against GrCh38 with RefSeq gene definitions. Adapted from Version 8.25 from the Textbook OpenStax Anatomy and Physiology Published May 18, 2016, licensed under the Creative Commons Attribution 4.0 International license.

These methods are expanded versions of descriptions in our related work titled “Subtle left-right asymmetry of gene expression profiles in embryonic and foetal human brains”^8^. A schematic overview of this data collection can be found in Fig. 1.

Six embryos were collected by the MRC/Wellcome-Trust funded Human Developmental Biology Resource (HDBR – www.hdbr.org) (United Kingdom) at either CS15 or CS16, therefore estimated between 5 and 5.5 weeks post conception. The embryos were obtained anonymously from voluntary medical terminations (a combination of mifepristone and misoprostol) or physical termination (according to the mother’s choice), following appropriate informed consent by the donors, and with ethical approval from the Newcastle and North Tyneside NHS Health Authority Joint Ethics Committee. Donors to HDBR are asked to give written consent for the embryonic material to be collected, and are only approached once a decision to terminate their pregnancy has been made. The abortions were not because of observed congenital malformations or suspected genetic disorders. Karyotypes were normal.

The development of the embryos was assessed and designated to the relevant Carnegie stage (CS)^9^, using a practical staging guide devised to enable staging to a particular CS and using the external morphology of a single sample^10^. Forebrain and midbrain were separated and then dissected into left and right sides. The dissections were performed by multiple people from a small 4 member group following the same dissection procedure.

RNA was extracted at HDBR Newcastle. Tissue samples were divided into sub-samples each weighing thirty milligrams, which was the maximum loading capacity of the columns of the RNA purification kits). The sub-samples were homogenised using a Precellys 24 bead mill homogeniser (Bertin Corp. Rockville, MD, USA) using ceramic 1.4 mm beads for soft tissue homogenising (CK14) with 600ul of RTL plus Buffer with 10 μl/ml of β-Mercaptoethanol and 5 μl/ml of reagent DX (Qiagen, Venlo, the Netherlands). RNA and DNA was extracted from the tissue with a QIAcube using an AllPrep DNA/RNA Mini Kit (Qiagen) following the manufacturer’s recommended protocol. RNA was then pooled from all 30 mg sub-samples belonging to a given embryo’s left or right midbrain, or left or right forebrain. RNA quality was assessed using an Agilent 2100 Bioanalyzer (Applied Biosystems, Santa Clara, CA, USA), and then RNA was shipped on dry ice to Beijing Genomics Institute (BGI) Shenzhen/HongKong, China (www.genomics.cn). The embryos were five males and one female. The female was from a physical pregnancy termination, the males from chemical pregnancy terminations.

At BGI, the RNA was treated with DNAse and quality was determined again on an Agilent 2100 Bioanalyzer. All samples passed the quality filters of: ≥ 4 μg RNA; concentration ≥ 80 ng/μL; RIN ≥ 7.0; 28 S/18 S ≥ 1.0; smooth baseline and normal 5 S peak. 200 ng of RNA was treated with Oligo-dT beads to enrich for mRNA out of the pool of total RNA. Afterwards, the purified RNA was broken into short segments in Fragment Buffer (New England Biolabs). After fragmentation, cDNA was generated with random hexamer primers, using First Strand Master Mix with Superscript II (Invitrogen) reverse transcriptase, and next the Second Strand Master Mix. End-repair Mix was added, and the repaired fragments were purified with AMPure(R) XP beads (Agencourt(R) ). A-tailing mix was added, then cDNA fragments were connected with adapters, following standard Illumina(R) procedures. Next followed again a round of purification with AMPure XP beads. To enrich the library further, several rounds of PCR were used, followed by a new round of purification. The final library was quantitated in two ways: the average molecule length was determined using the Agilent 2100 bioanalyzer instrument (Agilent DNA 1000 Reagents), and the library was quantified by real-time quantitative PCR (QPCR) (TaqMan Probe, Thermo Fisher Scientific). The libraries were now firstly amplified within the flow cell on the cBot instrument for cluster generation (HiSeq® 4000 PE Cluster Kit, Illumina). Then, the clustered flowcell was loaded onto the HiSeq 4000 Sequencer for paired-end sequencing (HiSeq® 4000 SBS Kit, Illumina) with read lengths of 100 bases.

### Data processing

At BGI, raw reads were filtered to exclude reads with more than 5% unknown bases, reads which contained more than 20% bases with quality score below 15, and reads with adapters. After filtering, the median size was 5.65 Gb per library (range 3.66–7.26 Gb). RNA sequencing data were produced as fastq-files by BGI. These files comprise the bulk of the current data release (Data Citation 1).

Sequence reads were then aligned to the Human reference GRCh38 from UCSC (http://genome-euro.ucsc.edu) using Hisat2 (v2.0.4). Using the same reference with RefSeq gene annotations, reads were then counted per gene using RSEM (v1.3.09). Both packages used bowtie2^11^. In R (version 3.3.2), expression data were normalized and transformed into log2 cpm (counts per million). From these processes we produced a file containing per gene log2 transformed read counts per sample, with genes indicated by Entrez IDs, which is also included in the current release (Data Citation 1). The report from BGI with details about the sequencing and mapping results is included as a supplement (Supplementary File 1). Figures 6 and 7 of this Supplementary File 1 show a satisfactory coverage of transcripts by the reads.

Finally, multidimensional scaling (MDS) analysis in R showed that forebrain and midbrain separated into two distinct clusters on the basis of overall gene expression similarity. However, the right side of one of the forebrain samples fell into the midbrain cluster. Data for the forebrain were therefore discarded for this embryo, and are excluded from the current release. In addition, both sides of one midbrain sample clustered with the forebrain group, and the midbrain samples for this embryo were discarded as well, and excluded from the current release. We are therefore releasing data from four males and one female for each structure, though not exactly the same embryos for forebrain and midbrain (See Table 1 for an overview of the samples).

Note that for the purposes of MDS analysis, and our other downstream analysis of this dataset which we report elsewhere, genes were additionally filtered to retain only those for which at least three libraries had at least five reads per gene, separately for forebrain and midbrain. However, this step has not been applied to the gene count table which we are releasing.

### Code availability

Codes that were used for processing the data are available as supplement (Supplementary File 2).

## Data Records

FASTQ sequencing files for six embryos times two brain structures times two sides, minus one forebrain and one midbrain = 20 libraries (2 files per library because of the paired reads), have been deposited to the Gene Expression Omnibus with series number GSE99302 (Data Citation 1). Individual accession numbers for each biological sample are also provided in Table 2 and with more details in Supplementary File 3 (Excel).

For users not interested in re-processing the data, a processed file of per gene log2 transformed read counts per sample, with genes indicated by Entrez IDs, is also included ‘GSE99302_BGI_2Tissues_expression_log2.txt.gz’ at NCBI Gene Expression Omnibus GSE99302. Per sample, text files are available with the following columns: gene_id (Entrez gene id), expected count (number of reads), FPKM (Fragments Per Kilobase Million), Symbol (Gene Symbol).

## Technical Validation

**Figure 2:**
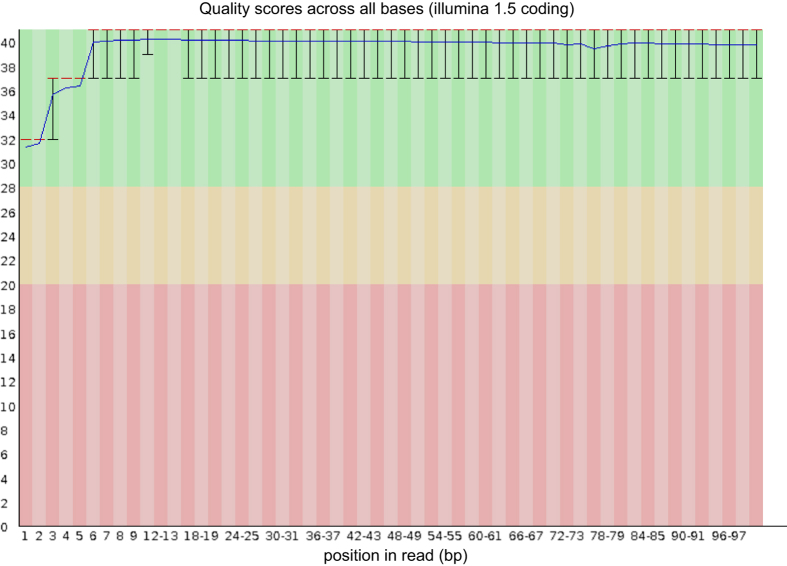
FastQC quality scores for a representative sample.

Descriptive analysis with the FastQC software (v0.11.5, Babraham Bioinformatics, Cambridge, USA) showed that the quality was high without overrepresented sequences or adapters, and phred base quality score mostly > 37 (Fig. 2). GC content varied between 48 and 50%.

A separate pipeline using HiSat (v0.1.6 beta, for mapping against hg19) and GATK (v3.4.0 (ref. 12)) was used at BGI to create genotype calls for single nucleotide polymorphisms (SNPs) from the RNA sequencing data. The SNP data were then used in plink (v1.07^13^) to confirm that left and right pairs of matched samples came from the same individual, and to confirm the sexes of the samples. A second confirmation for sex was found by looking at the expression data for the X-chromosomal gene *XIST* and the Y-chromosomal genes *EIF1AY* and *KDM5D*. *XIST* should be expressed in the female, while *EIF1AY* and *KDM5D* should not. Sex was confirmed, as well as proper matching of RNA from a given embryo.

**Figure 3:**
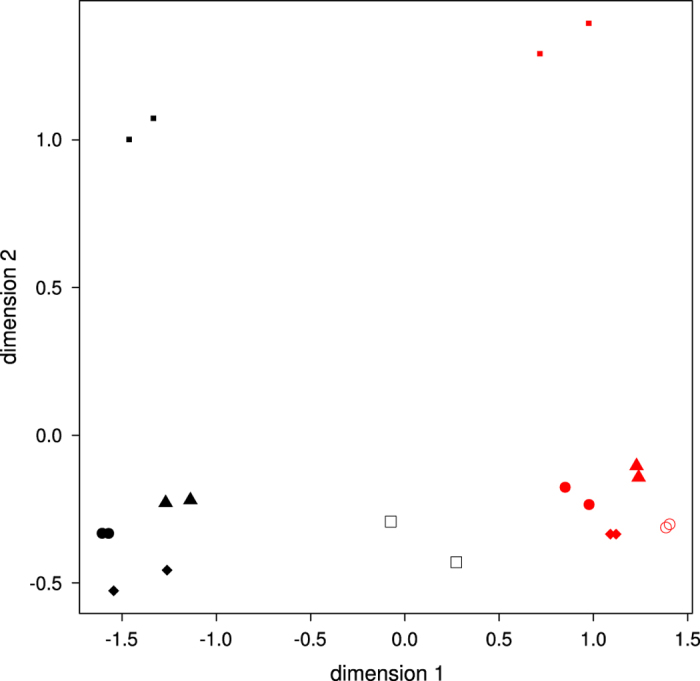
MDS-plot based on gene expression in midbrain and forebrain samples at 5-5.5pcw. Forebrain samples are shown in black, midbrain samples in red. Unique symbols refer to individual embryos. The smaller squares show the female embryo.

For our analysis of this dataset which we report elsewhere, MDS analysis was repeated using the expression data of the remaining five embryos per brain structure (after the aforementioned exclusions). The forebrain and midbrain clustered separately, and also the female was separated from the males, while left and right sides from the same embryo tended to cluster together (Fig. 3).

### Comparison with published studies

Expression levels per gene, for the forebrain and the midbrain, were compared to those from a publically available dataset in which left and right had not been divided: E-MTAB-4840^14^ in the ArrayExpress database (Data Citation 2). From this latter dataset, data for embryos in the ages 4 to 9 weeks were used. Data were available as fastQ files and were processed in the same way as described above. Across all genes, correlations of average expression per gene for our embryos (5–5.5 weeks old) with those in dataset E-MTAB-4840 (4–9 weeks old) were r = 0.84 for forebrain and r = 0.80 for midbrain.

## Additional information

**How to cite this article**: de Kovel, C. G. F. *et al*. Transcriptomic analysis of left-right differences in human embryonic forebrain and midbrain. *Sci. Data* 5:180164 doi: 10.1038/sdata.2018.164 (2018).

**Publisher’s note**: Springer Nature remains neutral with regard to jurisdictional claims in published maps and institutional affiliations.

## Supplementary Material



Supplementary File 1

Supplementary File 2

Supplementary File 3

## Figures and Tables

**Table 1:** Overview of samples.

ID	Age (pcw)	Sex	FB	MB
S13128	5	Male	Y	Y
S13048	5.5	Female	Y	Y
S13052	5.5	Male	Y	Y
S13097	5.5	Male	Y	N
S13192	5.5	Male	N	Y
S13290	5.5	Male	Y	Y
Y = released. N = not released (excluded after MDS clustering analysis). FB = forebrain. MB = midbrain. Age is given in post conception weeks (pcw).				

**Table 2:** Sequence data as FASTQ files deposited to the Gene Expression Omnibus with series number GSE99302.

Subject ID	Protocol 1	Protocol 2	Protocol 3	Data (GSE99302)
S13048	Forebrain dissection left	RNA extraction (poly-A)	RNA-Seq	GSM2640755
S13048	Forebrain dissection right	RNA extraction (poly-A)	RNA-Seq	GSM2640757
S13048	Midbrain dissection left	RNA extraction (poly-A)	RNA-Seq	GSM2640756
S13048	Midbrain dissection right	RNA extraction (poly-A)	RNA-Seq	GSM2640758
S13052	Forebrain dissection left	RNA extraction (poly-A)	RNA-Seq	GSM2640759
S13052	Forebrain dissection right	RNA extraction (poly-A)	RNA-Seq	GSM2640761
S13052	Midbrain dissection left	RNA extraction (poly-A)	RNA-Seq	GSM2640760
S13052	Midbrain dissection right	RNA extraction (poly-A)	RNA-Seq	GSM2640762
S13097	Forebrain dissection left	RNA extraction (poly-A)	RNA-Seq	GSM2640763
S13097	Forebrain dissection right	RNA extraction (poly-A)	RNA-Seq	GSM2640764
S13097	Midbrain dissection left	RNA extraction (poly-A)	RNA-Seq	dropped
S13097	Midbrain dissection right	RNA extraction (poly-A)	RNA-Seq	dropped
S13128	Forebrain dissection left	RNA extraction (poly-A)	RNA-Seq	GSM2640765
S13128	Forebrain dissection right	RNA extraction (poly-A)	RNA-Seq	GSM2640767
S13128	Midbrain dissection left	RNA extraction (poly-A)	RNA-Seq	GSM2640766
S13128	Midbrain dissection right	RNA extraction (poly-A)	RNA-Seq	GSM2640768
S13192	Forebrain dissection left	RNA extraction (poly-A)	RNA-Seq	dropped
S13192	Forebrain dissection right	RNA extraction (poly-A)	RNA-Seq	dropped
S13192	Midbrain dissection left	RNA extraction (poly-A)	RNA-Seq	GSM2640769
S13192	Midbrain dissection right	RNA extraction (poly-A)	RNA-Seq	GSM2640770
S13290	Forebrain dissection left	RNA extraction (poly-A)	RNA-Seq	GSM2640771
S13290	Forebrain dissection right	RNA extraction (poly-A)	RNA-Seq	GSM2640773
S13290	Midbrain dissection left	RNA extraction (poly-A)	RNA-Seq	GSM2640772
S13290	Midbrain dissection right	RNA extraction (poly-A)	RNA-Seq	GSM2640774
