# Supplementary material for: Transcriptomic analysis of left-right differences in human embryonic forebrain and midbrain
Source: Sci Data. 2018 Sep 4;5:180164. doi: 10.1038/sdata.2018.164 (PMC6122166; doi:10.1038/sdata.2018.164)
Supplement: Supplementary File 1 [file sdata2018164-s2.pdf]

HUMkvhE [Transcriptome Resequencing Report]

BGI Co., Ltd.

Thursday, 10th Nov., 2016

Table of Contents

Results 2
- 1 Abstract 2
- 2 Sequencing Reads Filtering 2
- 3 Genome Mapping 3
- 4 SNP and INDEL Detection 4
- 5 Gene Expression Analysis 6

Methods 9
- 1 Transcriptome Resequencing Study Process 10
- 2 Sequencing Reads Filtering 10
- 3 Genome Mapping 10
- 4 SNP and INDEL Detection 11
- 5 Gene Expression Analysis 11

Help 11
- 1 FASTQ Format 11
- 2 What is TF 12
- 3 RNA editing format 12
- 4 Gene fusion format 12
- 5 DSG format 12
- 6 Gene expression list format 13
- 7 DEG list format 13
- 8 MA plot 14
- 9 Volcano plot 14
- 10 Cluster list format 14
- 11 VCF format 14
- 12 How to read DEG GO enrichment analysis result 14
- 13 How to read DEG pathway enrichment analysis result 15

References 16

Results

1 Abstract

In our project, we sequence 24 samples use Illumina Hiseq platform, and on average we generated about 5.64 Gb bases from each sample. After mapping sequenced reads to reference genome and reconstruct transcripts,we finally get novel transcripts from all samples, of this, are previously unknown splicing event for known gene, are novel coding transcripts without any known features, and the remaining are long noncoding RNA.

2 Sequencing Reads Filtering

The sequencing reads which containing low-quality, adaptor-polluted and high content of unknown base(N) reads, should be processed to remove this reads before downstream analyses. After filtering, reads quality metrics are shown as **Table 1** . The distribution of base content and quality are shown as **Figure 1** and **Figure 2** , respectively.

Table 1 Summary of sequencing reads after filtering. (Download)

Sample	Total Raw Reads(Mb)	Total Clean Reads(Mb)	Total Clean Bases(Gb)	Clean Reads Q20(%)	Clean Reads Q30(%)	Clean Reads Ratio(%)
S13048-LFB	57.73	54.69	5.47	99.03	96.56	94.72
S13048-LMB	58.99	56.04	5.60	99.06	96.71	95.00
S13048-RFB	65.43	62.34	6.23	99.08	96.75	95.27
S13048-RMB	74.01	70.53	7.05	99.06	96.75	95.30
S13052-LFB	45.54	43.44	4.34	99.06	96.66	95.39
S13052-LMB	53.60	50.87	5.09	98.91	96.15	94.91
S13052-RFB	73.43	69.77	6.98	99.06	96.69	95.02
S13052-RMB	70.17	66.68	6.67	99.02	96.59	95.02
S13097-LFB	68.91	65.36	6.54	98.94	96.30	94.85
S13097-LMB	46.95	44.81	4.48	99.08	96.69	95.44
S13097-RFB	75.61	72.08	7.21	99.07	96.74	95.33
S13097-RMB	45.87	43.37	4.34	98.95	96.29	94.55
S13128-LFB	76.64	72.60	7.26	99.04	96.58	94.73
S13128-LMB	65.18	61.97	6.20	99.06	96.66	95.08
S13128-RFB	53.13	50.12	5.01	98.98	96.38	94.34
S13128-RMB	45.25	42.76	4.28	99.07	96.76	94.48
S13192-LFB	59.85	57.02	5.70	99.09	96.80	95.28
S13192-LMB	66.07	63.21	6.32	99.10	96.81	95.66
S13192-RFB	38.39	36.59	3.66	99.09	96.78	95.31
S13192-RMB	58.29	54.80	5.48	99.04	96.59	94.01
S13290-LFB	62.97	59.95	6.00	99.03	96.59	95.21
S13290-LMB	50.56	47.82	4.78	99.08	96.77	94.58
S13290-RFB	50.01	47.78	4.78	99.09	96.80	95.55
S13290-RMB	62.80	59.19	5.92	99.04	96.64	94.25

Q20: the rate of bases which quality is greater than 20.

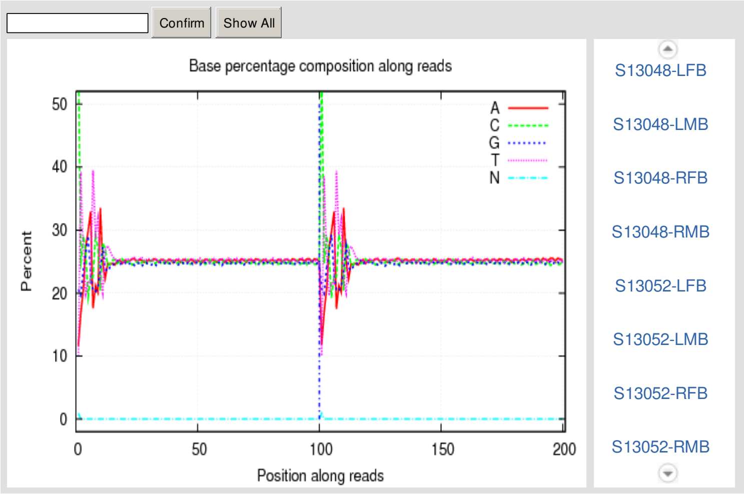

Figure 1 Distribution of base composition on clean reads. X axis represents base position along reads.Y axis represents base content percentage. As to high quality sequencing reads, A(adenine base) curve should be strictly overlapped with T(thymine base) curve and G(guanine bsase) curve should be overlapped with C(cytosine base) curve according to the principle of complementary of base pairing, excluding the first six base positions owing to Illumina sequencing platform using random hexamer-primer to synthesize cDNA which could result in PCR bias. As shown if figure, big fluctuations in first six base positons along reads, it is normal situation. If abnormal condition happens during sequencing, it may show an unbalanced composition.

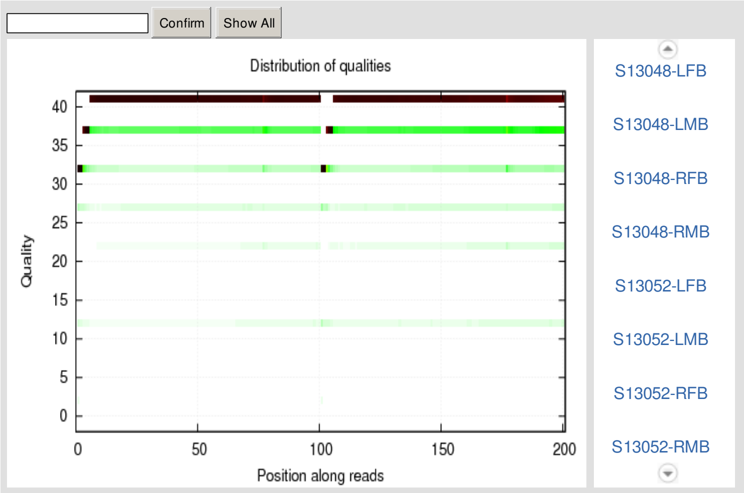

Figure 2 Distribution of base quality on clean reads. X axis represents base positions along reads. Y axis represents base quality value. Each dot in the image represents the number of total bases with certain quality value of the corresponding base along reads. Darker dot color means greater bases number. If the percentage of the bases with low quality (< 20) is very high, then the sequencing quality of this lane is bad.

3 Genome Mapping

After reads filtering, we map clean reads to reference genome use HISAT [1]. On average 94.59% reads are mapped, and the uniformity of the mapping result for each sample suggests that the samples are comparable. The mapping details are shown as **Table 2** .

Table 2 Summary of Genome Mapping (Download)

Sample	Total CleanReads	Total MappingRatio	Uniquely MappingRatio
S13048-LFB	54,686,510	94.63%	88.43%
S13048-LMB	56,042,480	94.57%	89.13%
S13048-RFB	62,340,440	94.88%	88.95%
S13048-RMB	70,529,788	94.91%	89.69%
S13052-LFB	43,441,728	94.66%	88.09%
S13052-LMB	50,868,912	94.75%	88.66%
S13052-RFB	69,774,640	94.54%	87.98%
S13052-RMB	66,677,290	94.78%	88.78%
S13097-LFB	65,362,962	94.27%	87.82%
S13097-LMB	44,808,580	94.26%	87.75%
S13097-RFB	72,082,168	94.78%	88.35%
S13097-RMB	43,373,624	94.44%	88.25%
S13128-LFB	72,600,782	94.10%	87.25%
S13128-LMB	61,969,498	94.74%	88.46%
S13128-RFB	50,115,902	93.88%	86.93%
S13128-RMB	42,755,678	94.90%	88.82%
S13192-LFB	57,021,658	94.52%	87.94%
S13192-LMB	63,205,554	94.58%	88.23%
S13192-RFB	36,586,752	94.55%	88.87%
S13192-RMB	54,802,924	94.55%	87.96%
S13290-LFB	59,953,920	94.73%	88.59%
S13290-LMB	47,822,376	94.76%	88.97%
S13290-RFB	47,780,758	94.78%	88.69%
S13290-RMB	59,189,404	94.60%	88.77%

Uniquely Mapping: Reads that map to only one location of reference, called uniquely mapping.

4 SNP and INDEL Detection

After genome mapping, we use GATK [2] to call ***SNP*** and ***INDEL*** variant for each sample. Final results are stored in VCF format. The ***SNP*** summary is shown as **Table 3** , and **Figure 3** . We also generate a friendly-interfaced ***SNP*** summary in EXCEL format shown as **Table 52** . And then we statistic the location of ***SNP*** and ***INDEL*** , shown as **Figure 4** and **Figure 5** .

Table 3 SNP variant type summary. (Download)

Sample	A-G	C-T	Transition	A-C	A-T	C-G	G-T	Transversion	Total
S13048-LFB	37,527	37,058	74,585	6,405	4,793	8,732	6,513	26,443	101,028
S13048-LMB	48,878	48,319	97,197	8,368	6,091	11,296	8,502	34,257	131,454
S13048-RFB	44,620	43,955	88,575	7,649	5,631	10,222	7,712	31,214	119,789
S13048-RMB	60,882	60,533	121,415	10,371	7,831	13,701	10,497	42,400	163,815
S13052-LFB	35,729	35,446	71,175	5,944	4,242	7,989	5,906	24,081	95,256
S13052-LMB	42,259	41,495	83,754	7,023	5,060	9,443	7,115	28,641	112,395
S13052-RFB	48,695	47,917	96,612	7,852	5,676	10,406	7,806	31,740	128,352
S13052-RMB	54,958	53,683	108,641	8,887	6,546	11,798	8,893	36,124	144,765
S13097-LFB	37,405	36,912	74,317	6,267	4,472	8,532	6,308	25,579	99,896
S13097-LMB	28,187	28,243	56,430	4,840	3,446	6,661	4,802	19,749	76,179
S13097-RFB	35,946	35,109	71,055	6,192	4,334	8,344	6,181	25,051	96,106
S13097-RMB	32,715	32,588	65,303	5,485	3,959	7,542	5,471	22,457	87,760
S13128-LFB	39,401	38,891	78,292	6,489	4,594	8,758	6,436	26,277	104,569
S13128-LMB	41,154	40,619	81,773	6,782	4,874	9,067	6,883	27,606	109,379
S13128-RFB	33,517	33,348	66,865	5,553	3,922	7,507	5,534	22,516	89,381
S13128-RMB	29,111	28,684	57,795	4,964	3,616	6,637	4,987	20,204	77,999
S13192-LFB	44,129	43,372	87,501	7,180	5,229	9,434	7,151	28,994	116,495
S13192-LMB	40,127	39,279	79,406	6,618	4,837	8,914	6,611	26,980	106,386
S13192-RFB	51,584	51,179	102,763	7,995	6,091	10,591	7,991	32,668	135,431
S13192-RMB	31,258	30,663	61,921	5,234	3,818	7,117	5,261	21,430	83,351
S13290-LFB	43,394	42,696	86,090	7,368	5,149	9,421	7,241	29,179	115,269
S13290-LMB	38,352	37,930	76,282	6,431	4,641	8,700	6,499	26,271	102,553
S13290-RFB	36,437	35,914	72,351	6,106	4,397	8,155	6,258	24,916	97,267
S13290-RMB	40,244	39,855	80,099	6,796	5,087	8,816	6,880	27,579	107,678

Transition: variant between purines or pyrimidines.Transversion: variant between purine and pyrimidine.

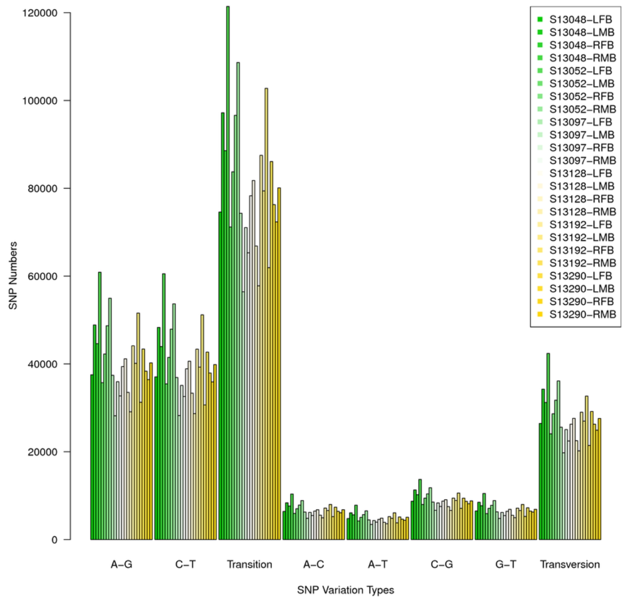

Figure 3 SNP variant type distribution. X axis represents the type of SNP. Y axis represents the number of SNP.

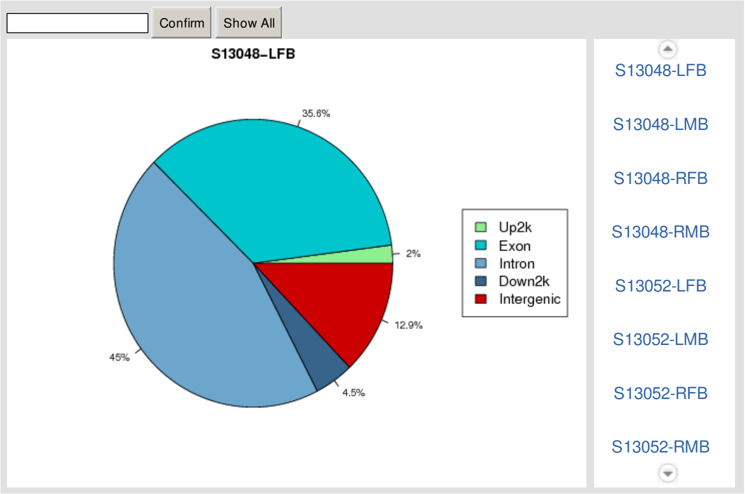

Figure 4 Distribution of SNP location. Up2k means upstream 2000 bp area of a gene. Down2k means downstream 2000 bp area of a gene.

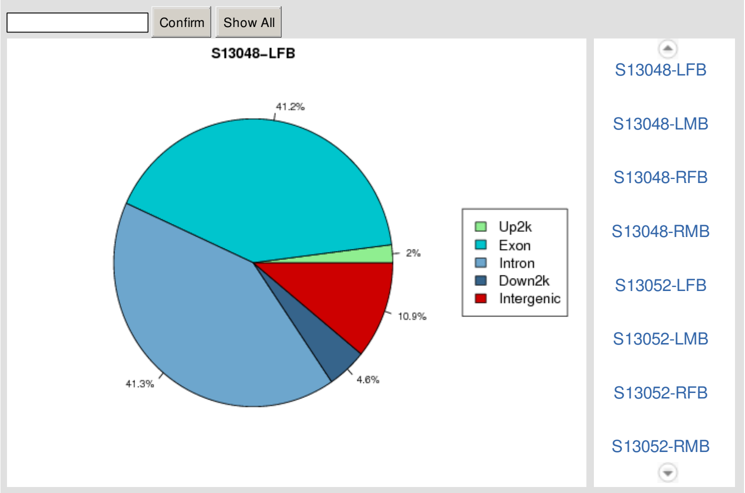

Figure 5 Distribution of INDEL location. Up2k means upstream 2000 bp area of a gene. Down2k means downstream 2000 bp area of a gene.

The VCF format ***SNP*** and ***INDEL*** result of each sample are shown as tables below(see VCF format in help page):

Table 4 SNP list of S13048-LFB (Download)

Table 5 SNP list of S13048-LMB (Download)

Table 6 SNP list of S13048-RFB (Download)

Table 7 SNP list of S13048-RMB (Download)

Table 8 SNP list of S13052-LFB (Download)

Table 9 SNP list of S13052-LMB (Download)

Table 10 SNP list of S13052-RFB (Download)

Table 11 SNP list of S13052-RMB (Download)

Table 12 SNP list of S13097-LFB (Download)

Table 13 SNP list of S13097-LMB (Download)

Table 14 SNP list of S13097-RFB (Download)

Table 15 SNP list of S13097-RMB (Download)

Table 16 SNP list of S13128-LFB (Download)

Table 17 SNP list of S13128-LMB (Download)

Table 18 SNP list of S13128-RFB (Download)

Table 19 SNP list of S13128-RMB (Download)

Table 20 SNP list of S13192-LFB (Download)

Table 21 SNP list of S13192-LMB (Download)

Table 22 SNP list of S13192-RFB (Download)

Table 23 SNP list of S13192-RMB (Download)

Table 24 SNP list of S13290-LFB (Download)

Table 25 SNP list of S13290-LMB (Download)

Table 26 SNP list of S13290-RFB (Download)

Table 27 SNP list of S13290-RMB (Download)

Table 28 INDEL list of S13048-LFB (Download)

Table 29 INDEL list of S13048-LMB (Download)

Table 30 INDEL list of S13048-RFB (Download)

Table 31 INDEL list of S13048-RMB (Download)

Table 32 INDEL list of S13052-LFB (Download)

Table 33 INDEL list of S13052-LMB (Download)

Table 34 INDEL list of S13052-RFB (Download)

Table 35 INDEL list of S13052-RMB (Download)

Table 36 INDEL list of S13097-LFB (Download)

Table 37 INDEL list of S13097-LMB (Download)

Table 38 INDEL list of S13097-RFB (Download)

Table 39 INDEL list of S13097-RMB (Download)

Table 40 INDEL list of S13128-LFB (Download)

Table 41 INDEL list of S13128-LMB (Download)

Table 42 INDEL list of S13128-RFB (Download)

Table 43 INDEL list of S13128-RMB (Download)

Table 44 INDEL list of S13192-LFB (Download)

Table 45 INDEL list of S13192-LMB (Download)

Table 46 INDEL list of S13192-RFB (Download)

Table 47 INDEL list of S13192-RMB (Download)

Table 48 INDEL list of S13290-LFB (Download)

Table 49 INDEL list of S13290-LMB (Download)

Table 50 INDEL list of S13290-RFB (Download)

Table 51 INDEL list of S13290-RMB (Download)

Table 52 Summary of population SNP (Download)

5 Gene Expression Analysis

After novel transcript detection, we merge novel coding transcripts with reference transcript to get complete reference, then we mapped clean reads to it use ***Bowtie2*** [3], then calculate gene expression level for each sample with ***RSEM*** [4]. The gene expression summary is shown as **Table 53** . And the gene expression list of each sample is shown as tables below(see Gene expression list format in help page).

We then calculate the reads coverage and the reads distribution on each detected transcript, shown as **Figure 6** and **Figure 7** , respectively. After that, we calculate pearson correlation between all samples, shown as **Figure 8** . Hierarchical clustering between all samples is also performed, shown as **Figure 9** .

Table 53 Summary of gene expression (Download)

Sample	Total CleanReads	Total MappingRatio	Uniquely MappingRatio	Total GeneNumber	Known GeneNumber	Novel GeneNumber	Total TranscriptNumber	Known TranscriptNumber	Novel TranscriptNumber
S13048-LFB	54,686,510	82.54%	34.25%	18846	18846	0	31219	31219	0
S13048-LMB	56,042,480	78.65%	32.63%	19235	19235	0	32143	32143	0
S13048-RFB	62,340,440	81.95%	34.14%	19139	19139	0	32037	32037	0
S13048-RMB	70,529,788	76.34%	31.38%	19563	19563	0	33180	33180	0
S13052-LFB	43,441,728	83.24%	34.52%	18589	18589	0	30333	30333	0
S13052-LMB	50,868,912	82.13%	33.91%	18889	18889	0	31195	31195	0
S13052-RFB	69,774,640	82.59%	34.30%	19181	19181	0	32263	32263	0
S13052-RMB	66,677,290	80.33%	33.14%	19321	19321	0	32579	32579	0
S13097-LFB	65,362,962	83.69%	35.27%	19011	19011	0	31880	31880	0
S13097-LMB	44,808,580	84.66%	35.76%	18677	18677	0	30410	30410	0
S13097-RFB	72,082,168	84.90%	35.74%	19163	19163	0	32434	32434	0
S13097-RMB	43,373,624	83.26%	35.11%	18740	18740	0	30447	30447	0
S13128-LFB	72,600,782	84.77%	35.08%	19135	19135	0	32363	32363	0
S13128-LMB	61,969,498	83.87%	34.68%	19026	19026	0	32120	32120	0
S13128-RFB	50,115,902	84.35%	34.95%	18773	18773	0	30897	30897	0
S13128-RMB	42,755,678	84.33%	34.49%	18478	18478	0	30050	30050	0
S13192-LFB	57,021,658	82.94%	34.31%	18839	18839	0	31323	31323	0
S13192-LMB	63,205,554	84.52%	35.27%	18882	18882	0	31637	31637	0
S13192-RFB	36,586,752	73.04%	30.04%	18679	18679	0	29979	29979	0
S13192-RMB	54,802,924	85.26%	35.31%	18704	18704	0	30914	30914	0
S13290-LFB	59,953,920	82.65%	34.50%	18904	18904	0	31430	31430	0
S13290-LMB	47,822,376	82.97%	34.58%	18714	18714	0	30688	30688	0
S13290-RFB	47,780,758	83.42%	34.84%	18583	18583	0	30530	30530	0
S13290-RMB	59,189,404	80.89%	33.03%	18849	18849	0	30992	30992	0

Uniquely Mapping: Reads that map to only one location of reference, called uniquely mapping.

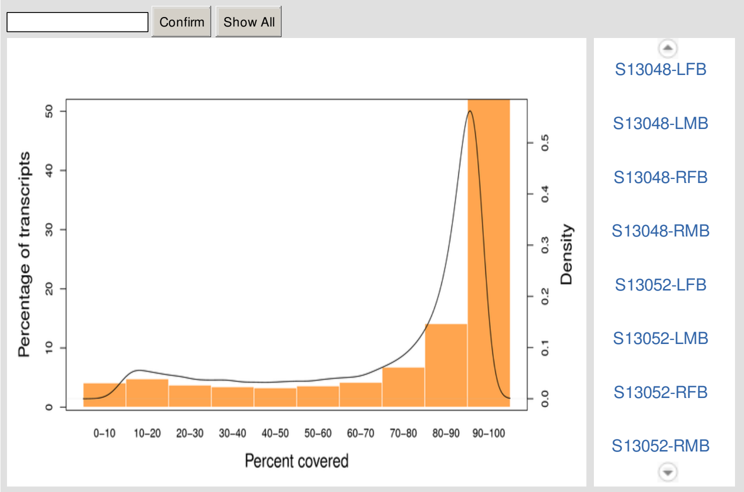

Figure 6 Reads coverage on transcripts. X axis represents the reads coverage. Y axis on left represents the percentage of transcripts. Y axis on right represents the density of transcripts.

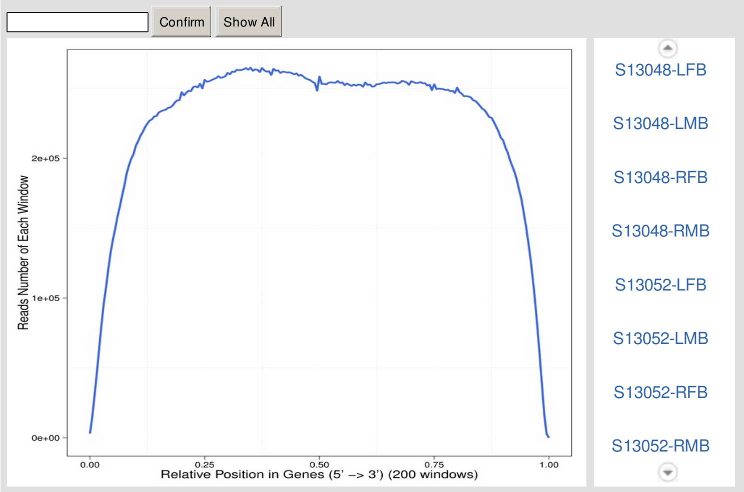

Figure 7 Reads distribution on transcripts. X axis represents the position along transcripts. Y axis represents the number of reads.

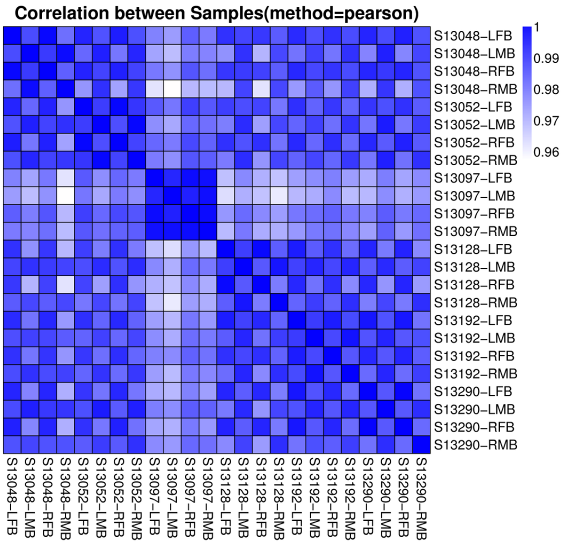

Figure 8 Heatmap of pearson correlation between samples. Both X and Y axis represent each sample. Coloring indicate pearson correlation(high: blue, low: white).

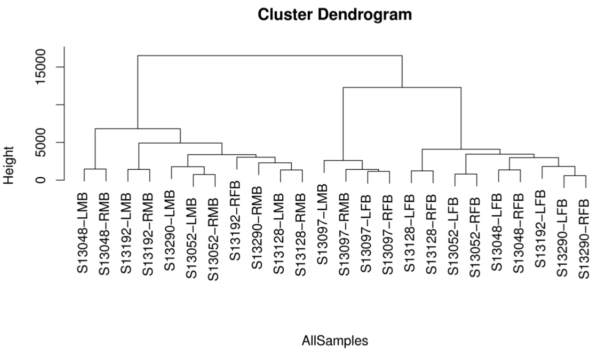

Figure 9 Hierarchical clustering between samples. More closer indicate more similar expression profile bewteen samples.

Table 54 Expressed gene list of S13048-LFB (Download)

Table 55 Expressed gene list of S13048-LMB (Download)

Table 56 Expressed gene list of S13048-RFB (Download)

Table 57 Expressed gene list of S13048-RMB (Download)

Table 58 Expressed gene list of S13052-LFB (Download)

Table 59 Expressed gene list of S13052-LMB (Download)

Table 60 Expressed gene list of S13052-RFB (Download)

Table 61 Expressed gene list of S13052-RMB (Download)

Table 62 Expressed gene list of S13097-LFB (Download)

Table 63 Expressed gene list of S13097-LMB (Download)

Table 64 Expressed gene list of S13097-RFB (Download)

Table 65 Expressed gene list of S13097-RMB (Download)

Table 66 Expressed gene list of S13128-LFB (Download)

Table 67 Expressed gene list of S13128-LMB (Download)

Table 68 Expressed gene list of S13128-RFB (Download)

Table 69 Expressed gene list of S13128-RMB (Download)

Table 70 Expressed gene list of S13192-LFB (Download)

Table 71 Expressed gene list of S13192-LMB (Download)

Table 72 Expressed gene list of S13192-RFB (Download)

Table 73 Expressed gene list of S13192-RMB (Download)

Table 74 Expressed gene list of S13290-LFB (Download)

Table 75 Expressed gene list of S13290-LMB (Download)

Table 76 Expressed gene list of S13290-RFB (Download)

Table 77 Expressed gene list of S13290-RMB (Download)

Methods

1 Transcriptome Resequencing Study Process

After extract total RNA and treated with DNase I, Oligo(dT) are used to isolate mRNA. Mixed with the fragmentation buffer, the mRNA are fragmented. Then ***cDNA*** is synthesized using the mRNA fragments as templates. Short fragments are purified and resolved with EB buffer for end reparation and single nucleotide A (adenine) addition. After that, the short fragments are connected with adapters. The suitable fragments are selected for the ***PCR*** amplification. During the QC steps, Agilent 2100 Bioanaylzer and ABI StepOnePlus Real-Time ***PCR*** System are used in quantification and qualification of the sample library. Then the library is sequenced using Illumina HiSeq 4000 or other sequencer when necessary.

After sequencing, we get raw reads. Firstly, we filter low-quality, adaptor-polluted and high content of unknown base(N) reads to get clean reads. And then mapping clean reads to reference genome, after that, novel transcript prediction ***SNP*** & ***INDEL*** detection differentially splicing gene(***DSG***) detection are performed. After we get novel transcripts, we merge conding transcripts of them with referecne transcript to get a complete reference, then we perform gene expression analysis with this reference. After that, we can detect Differentially Expression Gene(***DEG***) and perform further functional enrichment analysis between samples(two samples at least).Schematic overview of the comprehensive process is shown as **Figure 1** .

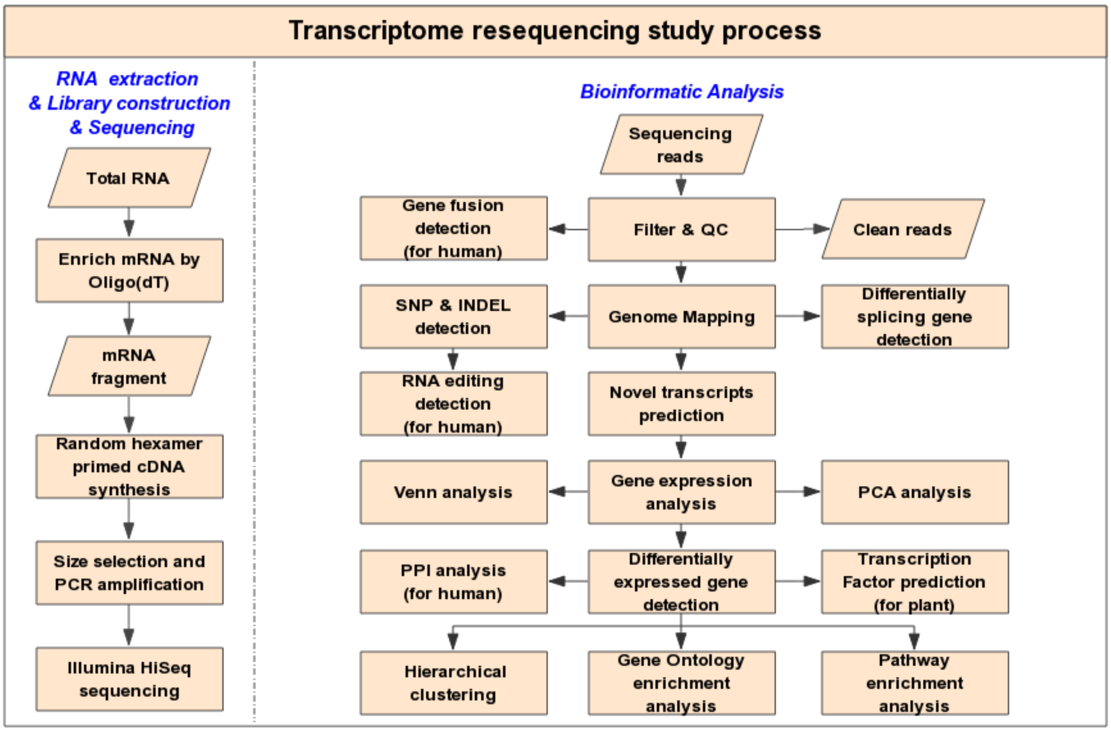

Figure 1 Transcriptome resequencing study process. Schematic overview of the study process.

2 Sequencing Reads Filtering

We define raw reads as reads which containing low-quality, adaptor-polluted and high content of unknown base(N) reads additionally, these noise reads should be removed before downstream analyses. We use internal software to filter reads, fllowed as:

1) Remove reads with adaptors;

2) Remove reads in which unknown bases(N) are more than 5%;

3) Remove low quality reads (we define the low quality read as the percentage of base which quality is lesser than 15 is greater than 20% in a read).

After filtering, the remaining reads are called "Clean Reads" and stored in FASTQ [8]format (see FASTQ Format in help page).

3 Genome Mapping

We use HISAT[1]to perfrom genome mapping, HISAT is a fast and sensitive spliced alignment program for mapping RNA-seq reads with equal or better accuracy than any other method. The paper show that, for simulated 20 million 100bp reads, the distribution of read types are shown as **Figure 2** , about 40% reads are spanning multiple exons, HISAT perform very well on this type reads.

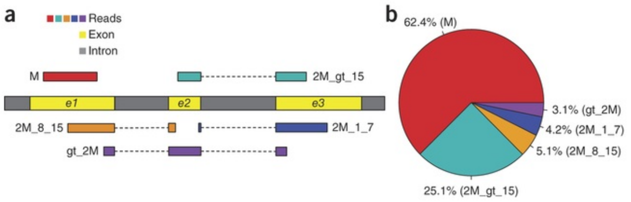

Figure 2 Distribution of read types. (a) Five types of RNA-seq reads: (i) M, exonic read; (ii) 2M_gt_15, junction reads with long, >15-bp anchors in both exons; (iii) 2M_8_15, junction reads with intermediate, 8- to 15-bp anchors; (iv) 2M_1_7, junction reads with short, 1- to 7-bp, anchors; and (v) gt_2M, junction reads spanning more than two exons. (b) Relative proportions of different types of reads in the 20 million 100-bp simulated read data.

Software information:

HISAT:
version: v0.1.6-beta
parameters: --phred64 --sensitive --no-discordant --no-mixed -I 1 -X 1000

4 SNP and INDEL Detection

With genome mapping result, we use GATK [2] to call ***SNP*** and ***INDEL*** for each sample. After filter out the unreliable sites, we get the final ***SNP*** and ***INDEL*** in VCF format. Software information:

GATK:
version: v3.4-0
parameters(call): -allowPotentiallyMisencodedQuals -stand_call_conf 20.0 -stand_emit_conf 20.0
parameters(filter): -window 35 -cluster 3 -filterName FS -filter "FS > 30.0" -filterName QD -filter "QD < 2.0"
website: https://www.broadinstitute.org/gatk

5 Gene Expression Analysis

we mapped clean reads to reference using ***Bowtie2*** [3], and then calculate gene expression level with ***RSEM*** [4]. ***RSEM*** is a software package for estimating gene and isoform expression levels from RNA-Seq data. With mapping result, we calculate reads coverage and reads distribution on transcripts. For a sample with hight quality and deep-enough depth, most of transcripts would be entirely covered, and mapped reads would be uniformly distributed on transcripts. After that, we calculate pearson correlation between all samples use cor, a function of R. After that, we perfrom hierarchical clustering between all samples use hclust, a function of R. Software information:

Bowtie2 **:**
version: v2.2.5
parameters: -q --phred64 --sensitive --dpad 0 --gbar 99999999 --mp 1,1 --np 1 --score-min L,0,-0.1 -I 1 -X 1000 --no-mixed --no-discordant -p 1 -k 200
website: http://bowtie-bio.sourceforge.net/ ***Bowtie2*** /index.shtml
RSEM **:**
version: v1.2.12
parameters: default
website: http://deweylab.biostat.wisc.edu/ ***RSEM***

Help

1 FASTQ Format

The original image data is transferred into sequence data via ***base calling*** , which is defined as raw data or raw reads and saved as FASTQ file. Those FASTQ files are the original data provided for users, including detailed read sequences and the read quality information. In each FASTQ file, every read is described by four lines, listed as follows:

```
@A80GVTABXX:4:1:2587:1979#ACAGTGAT/1
NTTTGATATGTGTGAGGACGTCTGCAGCGTCACCTTTATCGGCCATGGT
+
BMMTKZXUUUdddddddddddddddddddddddddddddadddddd^WYYU
```

The first and third lines are sequences names generated by the sequence analyzer; the second line is sequence; the fourth line is ***sequencing quality*** value, in which each letter corresponds to the base in line 2; the base quality is equal to ASCII value of the character in line 4 minus 64(we call the quality system is Phred+64), e.g. the

ASCII value of c is 99, then its base quality value is 35. Starting from the Illumina GA Pipeline v1.5, the range of base quality values is from 2 to 41. **Table 1** demonstrates the relationship between ***sequencing error*** rate and the ***sequencing quality*** value. Specifically, if the ***sequencing error*** rate is denoted as E and base quality value is denoted as Q, the relationship is as following formula:

$$SQ = -10 \times (\log\frac{E}{1-E})/(\log 10)$$

$$E = \frac{Y}{1+Y}$$

$$Y = \frac{SQ}{e^{-10 \times log10}}$$

Table 1 Relationship between sequencing error rate and sequencing quality value (Download)

Sequencing Error Rate(%)	Sequencing Quality Value	Character(Phred+46)	Character(Phred+33)
1.00	20	T	5
0.10	30	^	?
0.01	40	h	I

More detaild information about FASTQ format can be got in website http://en.wikipedia.org/wiki/FASTQ_format.

Note: The quality system of Illumina HiSeq 2000(or 2500) is Phred+64, and the quality system of Illumina HiSeq 4000 is Phred+33. For the reads sequencing by Illumina HiSeq 4000, in considering of the compatibility of softwares used in our study, we will convert the quality system from Phred+33 to Phred+64 for both raw data and clean data.

2 What is TF

In molecular biology and genetics, a transcription factor (sometimes called a sequence-specific DNA-binding factor) is a protein that binds to specific DNA sequences, thereby controlling the rate of transcription of genetic information from DNA to messenger RNA. Transcription factors perform this function alone or with other proteins in a complex, by promoting (as an activator), or blocking (as a repressor) the recruitment of RNA polymerase (the enzyme that performs the transcription of genetic information from DNA to RNA) to specific genes. See wiki for detail https://en.wikipedia.org/wiki/Transcription_factor.

3 RNA editing format

RNA editing list of each sample is stored in CNS format. See http://soap.genomics.org.cn/soapsnp.html Output Format for detail.

4 Gene fusion format

Gene fusion list of each sample is stored in tab-seperated text file. See http://soap.genomics.org.cn/soapfuse.html Output Files for detail.

5 DSG format

Differentially Splicing Gene(***DSG***) result of each compare plan is stored in tab-seperated text file Files/BGI_result/3.DifferentiallySplicingGene/*/*.*GeneDiffSplice.xls* with the format descriped in **Table 2** .

Table 2 Format of differentially splicing gene result list. (Download)

Field	Description	Notes
GeneID	gene identity	-
Chr	chromosome	-
Strand	strand	-
Control-IC	inclusion junction counts for Control sample, replicates are separated by comma	-
Control-SC	skipping junction counts for Control sample, replicates are separated by comma	-
Treat-IC	inclusion junction counts for Treat sample, replicates are separated by comma	-
Treat-SC	skipping junction counts for Treat sample, replicates are separated by comma	-
Pvalue	statistical significance	-
FDR	false discovery ratio	-
longExonStart	the long exon start position on chromosome	for A3SS and A5SS event
longExonEnd	the long exon end position on chromosome	for A3SS and A5SS event
shortExonStart	the short exon start position on chromosome	for A3SS and A5SS event
shortExonEnd	the short exon end position on chromosome	for A3SS and A5SS event
flankingExonStart	the flanking exon start position on chromosome	for A3SS and A5SS event
flankingExonEnd	the flanking exon end position on chromosome	for A3SS and A5SS event
1stExonStart	the first exon start position on chromosome	for MXE event
1stExonEnd	the first exon end position on chromosome	for MXE event
2ndExonStart	the secend exon start position on chromosome	for MXE event
2ndExonEnd	the secend exon end position on chromosome	for MXE event
riExonStart	the intron-retained exon start position on chromosome	for RI event
riExonEnd	the intron-retained exon end position on chromosome	for RI event
skipExonStart	the skipped exon start position on chromosome	for SE event
skipExonEnd	the skipped exon end position on chromosome	for SE event
upstreamExonStart	the upstream exon start position on chromosome	for RI and SE event
upstreamExonEnd	the upstream exon end position on chromosome	for RI and SE event
downstreamExonStart	the downstream exon start position on chromosome	for RI and SE event
downstreamExonEnd	the downstream exon end position on chromosome	for RI and SE event
LongExonTranscripts	the transcripts that contain long exon, separated by comma	for A3SS and A5SS event
ShortExonTranscripts	the transcripts that contain short exon, separated by comma	for A3SS and A5SS event
1stExonTranscripts	the transcripts that contain first exon, separated by comma	for MXE event
2ndExonTranscripts	the transcripts that contain secend exon, separated by comma	for MXE event
RetainTranscripts	the transcripts that contain intron-retained exon, separated by comma	for RI event
AbandonTranscripts	the transcripts that exclude intron-retained exon, separated by comma	for RI event
InclusionTranscripts	the transcripts that include certain exon, seperated by comma	for SE event
SkippingTranscripts	the transcripts that exclude certain exon, seperated by comma	for SE event

6 Gene expression list format

Gene expression result of each sample is stored in tab-seperated text file Files/BGI_result/4.Quantify/GeneExpression/**.gene.fpkm.xls*(* presents sample name) with the format descriped in **Table 3** .

Table 3 Format description of gene expression result list. (Download)

Field	Description
gene_id	gene ID number
transcript_id(s)	trascript list of gene, seperated by comma
length	length of gene after model regulation
expected_count	support reads number to this gene after model regulation
FPKM	FPKM value of this gene

7 DEG list format

The result of differentially expressed genes for each control-treatment pairwise is stored in tab-seperated text file Files/BGI_result/5.Quantify/DifferentExpressedGene/**.GeneDiffExpFilter.xls*(* presents pairwise name) with the format description in **Table 4** .

Table 4 Format description of DEGs screening result file. (Download)

Field	Description
Unigene	Unigene ID
Length	Unigene length
Sample1-Expression	Unigene expression of control sample(s)
Sample2-Expression	Unigene expression of treat sample(s)
log2FoldChange(Sample2/Sample1)	log2 transformed fold change between control and treat samples
Pvalue	Statistic of pvalue(PossionDis or DEseq2 method used)
FDR	Statistic of false discovery rate(PossinoDis method used)
Padj	Statistic of adjusted pvalue(DEseq2 method used)
PPEE	Statistic of posterior probability of being equivalent expression(EBseq method used)
Probability	Statistic of probability of being DEG(NOIseq method used)
Up/Down-Regulation(Sample2/Sample1)	Flags indicate up-regulated DEG(Up) or down-regulated DEG(Down) or non-DEG(*)

8 MA plot

The MA plot is a plot of the distribution of the red/green intensity ratio ('M') plotted by the average intensity ('A'). M and A are defined by the following equations:

$$M = \log_2(R/G) = \log_2(R) - \log_2(G)$$
$$A = \frac{1}{2}\log_2(RG) = \frac{1}{2}(\log_2(R) + \log_2(G))$$

See wiki for detail https://en.wikipedia.org/wiki/MA_plot.

9 Volcano plot

The Volcano plot is a type of scatter-plot that is used to quickly identify changes in large datasets, It plots significance versus fold-change on the y- and x-axes, respectively. See wiki for detail https://en.wikipedia.org/wiki/Volcano_plot_(statistics).

10 Cluster list format

The format of cluster list is described as **Table 5** .

Table 5 Format description of DEGs clustering list. (Download)

Field	Description
Unigene	Unigene ID
A-VS-B	log2FoldChange of A-VS-B
C-VS-D	log2FoldChange of C-VS-D
...	...

11 VCF format

Variant Call Format (VCF) is a flexible and extendable format for variation data such as single nucleotide variants, insertions/deletions, copy number variants and structural variants. See details at UCSC website http://genome.ucsc.edu/FAQ/FAQformat.html#format10.1

12 How to read DEG GO enrichment analysis result

Make sure that the computer has installed java and use IE brower to open *GOView.html.* The left navigation includes three types of GO terms for each control-treatment pairwise (C: cellular component, P: biological process, F: molecular function). Click one of them, the enriched GO terms result will be listed as **Figure 3** .

Gene Ontology term	Cluster frequency	Genome frequency of use	Corrected P-value	Expression Profile
BLOC complex (view genes)	2 out of 82 genes, 2.4%	8 out of 16090 genes, 0.0%	0.03943	View Result
cytosol (view genes)	2 out of 82 genes, 2.4%	15 out of 16090 genes, 0.1%	0.14450	View Result
cytosolic part (view genes)	2 out of 82 genes, 2.4%	15 out of 16090 genes, 0.1%	0.14450	View Result
intracellular part (view genes)	67 out of 82 genes, 81.7%	11513 out of 16090 genes, 71.6%	1	View Result

Figure 3 Significantly enriched GO terms in DEGs. Column 1 is GO term name. Column 2 is the ratio of DEGs enriched to this GO term. Column 3 is the ratio of genes enriched to this GO term in background database. Column 4 is Corrected P-value which indicates the degree of enrichment and the smaller Corrected P-value, the more significantly DEGs enriched to this GO term. The result list has been sorted by Corrected P-value. Column 5 is clustering of foldchange value for these enriched DEGs using the tools cluster [5] [6] and javaTreeView [7].

Click the term name 'BLOC complex' in **Figure 3** , you can go to http://amigo.geneontology.org/amigo for more information when the computer is Internet-connected. Click 'view genes' in **Figure 3** , you can get gene IDs that enriched to this GO term as **Figure 4** .

BLOC complex	63915, 100526837
cytosol	63915, 100526837

Figure 4 Gene ID list related to GO terms. There are two DEGs enriched to the term 'BLOC complex': 63915, 100526837.

13 How to read DEG pathway enrichment analysis result

Open html report for pathway enrichment result and the enriched KEGG pathways will be listed as **Figure 5** .

1. sample3-VS-sample4

#	Pathway	DEGs with pathway annotation (1432)	All genes with pathway annotation (17252)	Pvalue	Qvalue	Pathway ID
1	Pathways in cancer	81 (5.66%)	531 (3.08%)	5.562454e-08	1.074132e-05	ko05200
2	Focal adhesion	74 (5.17%)	475 (2.75%)	8.877128e-08	1.074132e-05	ko04510
3	Leukocyte transendothelial migration	46 (3.21%)	280 (1.62%)	5.86161e-06	3.950743e-04	ko04670
4	Rheumatoid arthritis	25 (1.75%)	115 (0.67%)	6.530153e-06	3.950743e-04	ko05323
5	Malaria	19 (1.33%)	76 (0.44%)	1.00329e-05	4.855924e-04	ko05144

Figure 5 Pathway enrichment analysis of DEGs. Column 1 is ordinal number. Column 2 is pathway name. Column 3 is the ratio of DEGs enriched to this pathway. Column 4 is the ratio of genes enriched to this pathway in background database. Pvalue and Qvalue are both values that indicate the degree of enrichment and Qvalue is corrected Pvalue. The smaller they are, the more significantly DEGs enriched to this pathway. The result list has been sorted by Qvalue. The last column pathway ID corresponding to pathway name.

Click pathway name 'Leukocyte transendothelial migration' in **Figure 5** , you can get gene IDs that enriched to it as **Figure 6** .

3	Leukocyte transendothelial migration	146850, 654463, 5909, 4318, 1364, 402415, 3383, 2888, 100528016, 5175, 9404, 149461, 285590, 5880, 50507, 79778, 58494, 8572, 8481, 6525, 5603, 90799, 55691, 100506649, 29970, 4739, 6876, 55679, 5010, 9076, 9411, 26509, 9758, 10398, 8727, 7412, 7070, 6387, 8502, 7430, 7414, 71, 60, 4771, 80014, 51306
4	Rheumatoid arthritis	2921, 6364, 6374, 3576, 3553, 4319, 2920, 2919, 3552, 4314, 2353, 4312, 3589, 100288077, 3383, 7099, 7422, 1514, 7040, 533, 7042, 6387, 284, 5157, 6347

Figure 6 Gene ID list related to pathway. There are 46 DEGs enriched to the pathway 'Leukocyte transendothelial migration'.

Furtherly, detecting the most significant pathways, the enrichment analysis of ***DEG*** pathway significance, allows us to see detailed pathway information in KEGG database. For example, clicking the hyperlink on 'Leukocyte transendothelial migration' in **Figure 6** will get detailed information as shown in **Figure 7** .

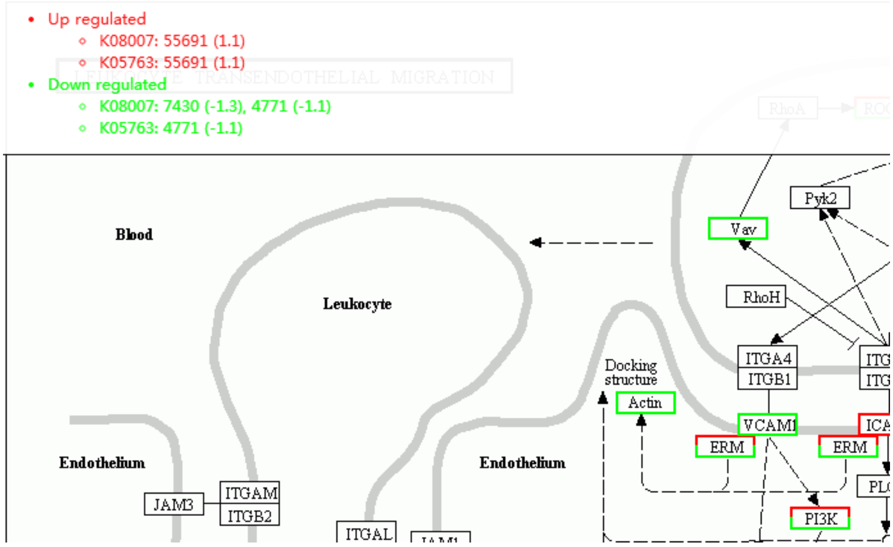

Figure 7 An example of KEGG pathway of 'Leukocyte transendothelial migration'. Up-regulated genes are marked with red borders and down-regulated genes with green borders. Non-change genes are marked with black borders. When mouse hover on border with red or green, the related DEGs appear on the top left. Clicking gene name in the figure, the page will redirect to KEGG website if the computer is Internet-connected.

References

[1] Kim D, et al.(2015).HISAT: a fast spliced aligner with low memory requirements. Nature Methods 2015.
[2] McKenna A, et al.(2010).The Genome Analysis Toolkit: a MapReduce framework for analyzing next-generation DNA sequencing data.Genome Res. 2010 Sep;20(9):1297-303.
[3] Langmead B, et al.(2012).Fast gapped-read alignment with Bowtie 2. Nature Methods. 2012, 9:357-359.
[4] Li B, et al.(2011).RSEM: accurate transcript quantification from RNA-Seq data with or without a reference genome.BMC Bioinformatics. 2011 Aug 4;12:323.
[5] Eisen, M. B., et al. (2001). Cluster analysis and display of genome-wide expression patterns. Proc Natl Acad Sci USA, (1998)95(25): 14863-8. 2001.29: 1165-1188.
[6] M. J. L. de Hoon, et al. (2004). Open Source Clustering Software.Bioinformatics, 20(9): 1453-1454.
[7] Saldanha, A. J. (2004). Java Treeview--extensible visualization of microarray data. Bioinformatics, 20(17): 3246-8.
[8] Cock P., et al.(2010). The Sanger FASTQ file format for sequences with quality scores, and the Solexa/Illumina FASTQ variants. Nucleic Acids Research, 38(6): 1767-1771.

2015 Copyright BGI All Rights Reserved 粤ICP备 12059600 Technical Support E-mail:info@bgitechsolutions.com Website: www.bgitechsolutions.com
